# Controlled sol–gel synthesis of oxygen sensing CdO : ZnO hexagonal particles for different annealing temperatures

**DOI:** 10.1039/c9ra05998a

**Published:** 2019-10-02

**Authors:** Jeevitesh K. Rajput, Trilok K. Pathak, Vinod Kumar, H. C. Swart, L. P. Purohit

**Affiliations:** Semiconductor Research Lab, Department of Physics, Gurukula Kangri University Haridwar India proflppurohitphys@gmail.com lppurohit@gkv.ac.in; Department of Physics, University of the Free State Bloemfontein South Africa; Centre for Energy Studies, Indian Institute of Technology Delhi New Delhi India

## Abstract

CdO : ZnO hexagonal particles were synthesized by a sol–gel precipitation method at different annealing temperatures. A mixed crystal phase of cubic and wurtzite structures was observed from X-ray diffraction patterns. The micrographs showed hexagonal shapes of the CdO : ZnO nanocomposites particles. The energy dispersive X-ray spectroscopy mapping images showed a uniform distribution of the Cd and Zn. The CdO : ZnO nanocomposite pallet annealed at 550 °C has an electrical resistance of 0.366 kΩ at room temperature. The nanocomposites showed an excellent sensing response against oxygen gas with a sensing response of 47% at 200 °C for the CdO : ZnO particles annealed at 550 °C. The sensor response and recovery times were found to be 43s and 45s, respectively. The sensor response was due to the sorption of oxygen ions on the surfaces of the CdO : ZnO hexagonal particles.

## Abstract 2

CdO : ZnO hexagonal particles were synthesized by a sol–gel precipitation method at different annealing temperatures.

## Graphical abstract

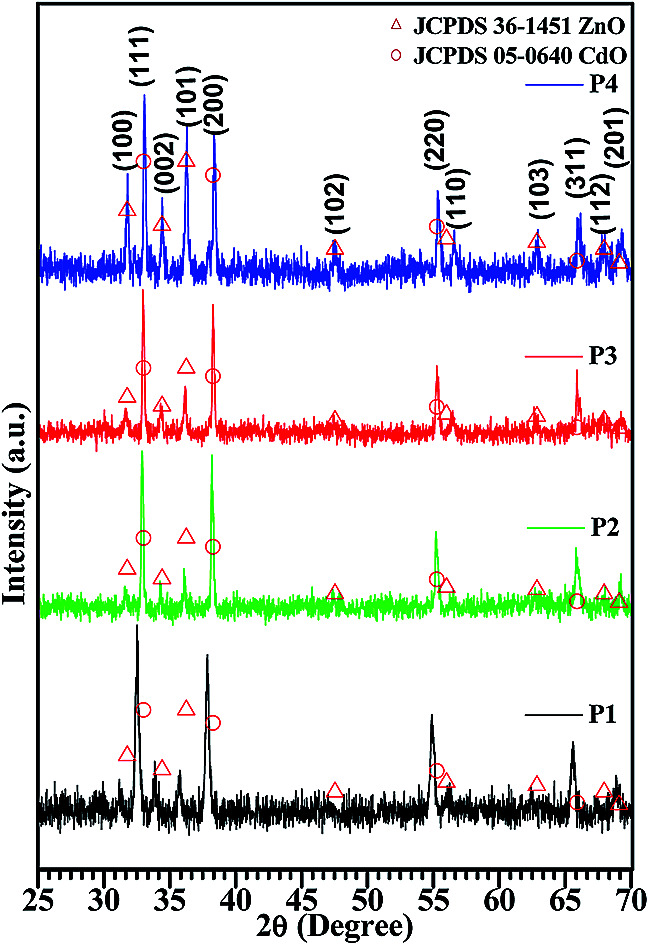

## 1. Introduction

At the present time, with the rapid increase of many demands to boost productivity and performance, not only in industry, but also in the domestic and agricultural settings, an increasing number of toxic, flammable and combustible chemicals and gases have been produced in our environment.^1^ The pollutants can be poisonous gases, pesticides, herbicides, fungicides, noise, and organic and by-product compounds such as pollutants that are increasing significantly and endangering human life. In the last decade, research has concentrating on developing devices to control and detect these pollutants. In order to fight gas pollutant issues researchers developed sensing devices for different toxic/non-toxic gases such as NH_3_, NO_2_, CO, H_2_S, liquid petroleum gas, O_2_ and N_2_.^2–8^ It's becoming very important to develop a detector for gases due to specific dangerous physical properties such as being colourless, odourless and tasteless, and thus undetectable by our human senses. Oxygen sensing and leakage from industrial installations are considered a critical problem to be resolved related to human health, aquatic life, and environmental issues. Oxygen leakage may lead to fire and explosion hazards in hyperbaric chambers used in medical treatment, food preservation and packing, steel works, and chemical plants.^8^ Several oxygen sensors were reported based on different oxides such as cadmium titanate oxides, and iron doped/undoped strontium titanate oxide, while limited reports are published for CdO–ZnO nanocomposite particles for oxygen sensing with such a rapid sensing response.^8–12^

One and two-dimensional nanostructures are extensively studied due to their peculiar and superior transport properties.^13^ Three-dimensional nanostructures such as nanosphere, nanocubes, hexagonal also showed promising application due to their large surface area.^14^ For the semiconducting sensing devices the surface area, size, and shape of nanostructures play a crucial role to improve the sensing properties of the electrochemical sensor. Metal oxide semiconductors are interestingly studied for above purposes. Rossinyol^15^ and his co-workers proposed a better control of the performance of tungsten oxide based 2D hexagonal nanostructures as sensing materials. Postica *et al.*^16^ reported the advantage of a zinc oxide tetrapod network microstructure in the gas sensing technology with his co-authors. ZnO, which has a large direct optical band gap (E_g_) of 3.2 eV with a hexagonal lattice is an important n-type semiconductor along with SnO_2_ and TiO_2_. In 2002, Delgado^17^*et al.* synthesised (CdO)_*y*_(ZnO)_1−*y*_ by a sol–gel process and revealed the remarkable controlled variation in structural, optical and electrical properties. The CdO is also an n-type metal oxide semiconductor with a similar electronic configuration to ZnO, while this have direct (2.2 eV) and indirect (1.98 eV) optical bandgaps with cubic and rock salt crystal lattices. In a controlled chemical synthesis several parameter such as concentration, pH value, dopant, annealing temperature, *etc.* affect the properties of devices.^18^ Among these parameters the annealing temperature has the strongest impact on the structure and morphology of the nanostructures. Imran *et al.*^8^ investigated the change in surface area and grain size of cadmium titanate nanofibers as function of annealing temperature and improved oxygen sensing.

In the present work, the effect of annealing on the structural, morphological and electrical properties of CdO : ZnO nanostructure synthesis by a sol–gel method was investigated. The sensing properties such as selectivity, sensor response, response time and recovery speed of the gas sensor were investigated in detail for the CdO : ZnO nanostructures and a possible sensing mechanism is proposed. Therefore for this study we can understand the role of annealing temperature on different properties of CdO : ZnO particles and this is also proposed a very low cost and easy method to obtain hexagonal particles, which are useful in surface reaction applications such as gas sensing. Furthermore, the obtained CdO : ZnO nanocomposites show excellent oxygen sensing properties.

## 2. Experimental details

### 2.1 Synthesis of CdO : ZnO hexagonal particles

The chemical precipitating method was used to synthesized CdO : ZnO hexagonal particles at different annealing temperature. The analytical grade chemicals were used as source materials likewise cadmium acetate dehydrate (CdAc, Sigma Aldrich) and zinc acetate dehydrate (ZnAc, Alfa Aesar) for cadmium (Cd) and zinc (Zn), respectively. Two separate solutions of similar concentration were prepared for the nanocomposites by dissolving CdAc (13.326 g) and ZnAc (10.974 g) in distilled water (100–100 ml). The two solutions were spun together in 3 : 1 volume ratio of CdO : ZnO solutions for 2 h at room temperature in laboratory conditions. After the aging of 24 h, the sodium hydroxide (NaOH) was added dropwise as precipitating reactant. The solid part was filtered from solution then washed several time by methanol and dried at 120 °C in a microprocessor controlled furnace for 4 h. To study the effect of annealing temperature the final products were annealed in an air ambiance to crystallize the oxides at four different annealing temperatures (ATs) 450, 500, 550 and 600 °C for 4 h in a furnace. Here after the CdO : ZnO particles were named sample P1, P2, P3 and P4.

### 2.2 Characterization of CdO : ZnO hexagonal particles

The structural properties were investigated by X-ray diffraction (XRD) with a Bruker D8 advance diffractometer with CuK_α_ radiation (wavelength 0.15418 nm). The morphology and elemental study of the samples were examined using scanning electron microscopy (SEM) and energy dispersive X-ray spectroscopy (EDS) with an EVO-40 ZEISS operated at an acceleration voltage of 20.0 kV. The current–voltage (*I*–*V*) measurements were carried out using a Keithley 4200-SCS electrometer. Relative humidity of the laboratory was measured by using a humidity sensor of Envirotech Instrument Private Limited.

The gas sensing performance of the CdO : ZnO hexagonal particles were studied in a specially designed gas sensor test rig (GSTR) having a sealed stainless steel cylindrical test chamber of 2.6 L volume. Pallets with a 1 cm diameter and 4 mm height were made for each sample. Two silver electrodes were coated on the pallet to measure electrical resistance of the sensors. The oxygen gas was allowed to flow through a gas needle valve from a gas flow meter into the chamber from a gas cylinder. For removal of any foreign gas the vacuum of the order of ∼10^−2^ torr (0.133 hPa) was first created in the test chamber and then the oxygen gas was introduced. Electrical measurements of the sensor were measured at a constant dc voltage of 1 V. The sensor was placed in the chamber under air atmosphere for measuring the sensor resistance in air (*R*_a_) and then the air was flushed out by using a rotary pump. The oxygen gas was introduced for measuring the corresponding sensor resistance in gas (*R*_g_) after attaining a stable resistance. The relative humidity (RH) of the laboratory and test chamber at air conditions was ∼60%. The rest of the parameters such as humidity, other gasses *etc.*, were kept constant throughout the experiment. The sensor response was calculated by using the well-known gas response formula (eqn (1))1
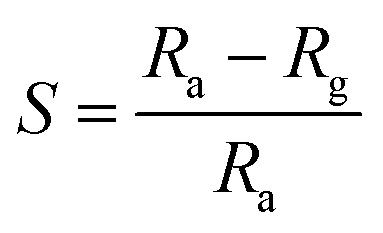
where, *R*_a_ and *R*_g_ are the resistance of the sensor in atmospheric air and in the presence of the target gas at a 1013 hPa pressure, respectively.^19^

## 3. Results and discussion

### 3.1 X-ray diffraction

**Fig. 1:**
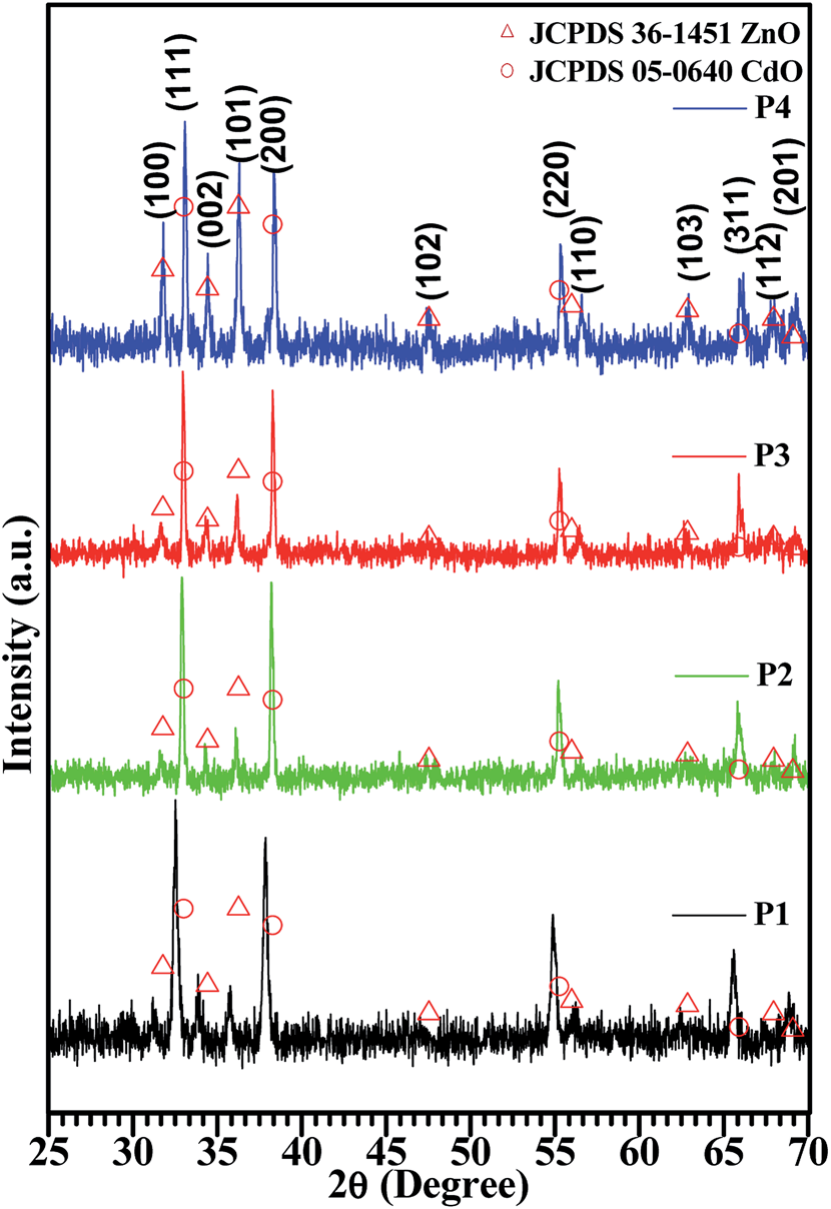
XRD spectra of CdO : ZnO NPs grew at different annealing temperatures and compared with JCPDS PDF# 04-0640 and PDF# 14-3651. The samples P1, P2, P3 and P4 were annealed at temperatures of 450 °C, 500 °C, 550 °C and 600 °C, respectively.

The crystal structure of the CdO : ZnO particles consisted out of two phases, *i.e.* cubic crystalline CdO as the host and the wurtzite hexagonal ZnO that was introduced as the modifier, as shown in Fig. 1. Standard JCPDS cards (PDF#05-0640, PDF#14-3651) of CdO and ZnO, were used to identify the phase composition and crystal structure of the annealed samples (P1–P4), by determining the relative peak positions and intensity ratios of the peaks in the different patterns. All the samples showed diffraction peaks corresponding to cubic CdO and hexagonal ZnO with a dominating cubic structure, which confirmed the majority of CdO in the mixture. Crystallization of the oxides was a strong function of ATs. The intensity of the diffraction peaks that corresponds to the wurtzite ZnO increased with the annealing temperature from 450 to 600 °C, which indicated that the higher AT was better for the formation of ZnO. This analysis shows that a higher thermal energy is required for the growth of the ZnO crystallites, while the CdO can grow at the lower temperatures as compared to the ZnO, because the lattice energy of the CdO (3806 kJ mol^−1^) is smaller than that of ZnO (4142 kJ mol^−1^).^4^ The position of the diffraction peaks are aligned with the standard peak positions for the high AT of sample P4. The intense peaks observed are centred at 33.09° and 38.38° with indices (111) and (200). This improvement may be attributed to a rearrangement of atoms and the removal of the defects with an increasing annealing temperature. The three other intense peaks were observed centred at 31.77°, 34.42° and 36.27°and indices to (100), (002) and (101), respectively, which confirmed the wurtzite hexagonal arrangement in the Zn^2+^ and O^2−^ atoms.

The crystals sizes calculated by Scherrer's formula by using the full width half maxima (FWHM) that were found by Gaussian peak fitting. The average values of the crystallite size were found to be 27 nm, 45 nm, 41 nm, 39 nm for sample P1–P4, respectively. It's observed that the maximum crystallite size was obtained for the middle range of the ATs. A similar result was observed for CdO nanostructures in our previous work.^20^ This variation can be related to the release of the in-plane compressive stress in the crystal. The present analysis confirms that the highly oriented crystals with smaller size were observed for the higher ATs.

### 3.2 Scanning electron microscopy

**Fig. 2:**
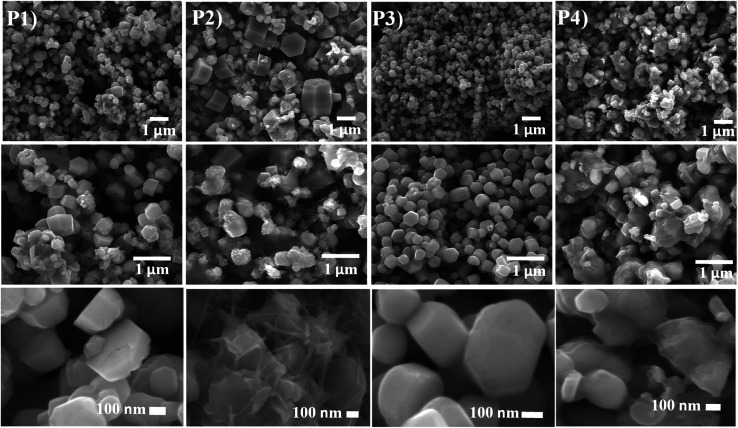
SEM images of ZnO:CdO NPs annealed at 450 °C (P1), 500 °C (P2), 550 °C (P3) and 600 °C (P4) with different scales.

**Fig. 3:**
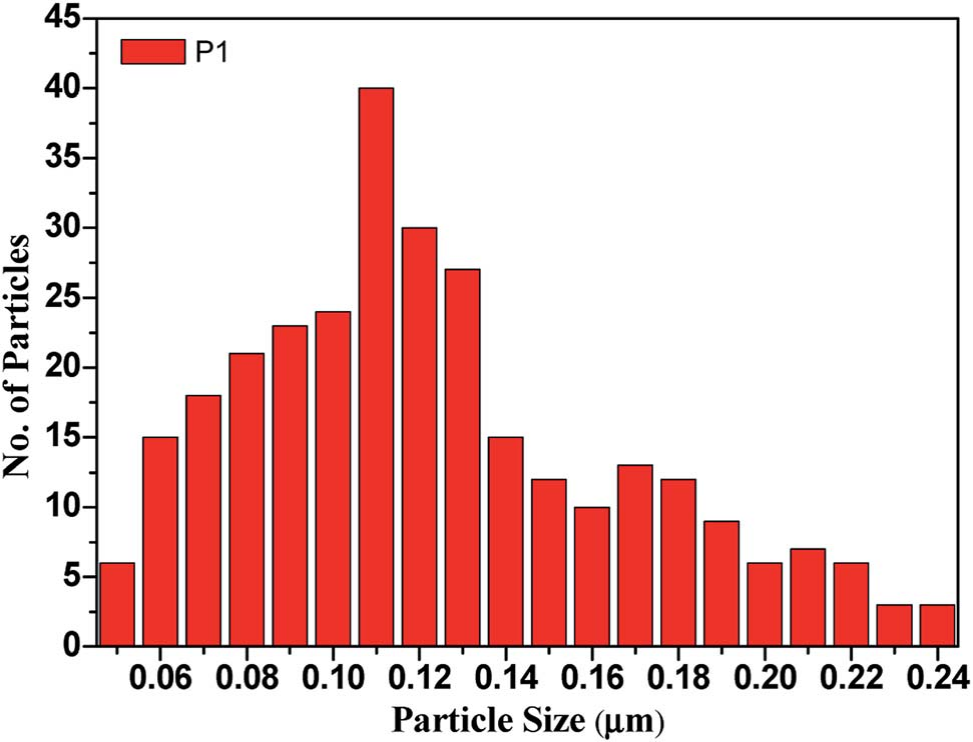
The bar diagram for determining the distribution of the particle size of the CdO : ZnO nanoparticles annealed at 450 °C using the ImageJ software.

The growth of the CdO : ZnO NPs were analysed by using SEM images as shown in Fig. 2. Three images were taken for each sample with different magnifications as indicated for the detail study of the NPs morphology. The low magnification pictures show uniform growth of an array of hexagonally packed particle of CdO : ZnO, while the high magnification micrographs show the individual structures of the particles more clearly. The size of the highly orientated hexagonal particles were measured by using ImageJ software and was found to be 140 nm in size as shown in Fig. 3. Sample P1, which was annealed at the lowest AT, formed smaller particles with a lower density. Sample P2 was grown in a more thermal environment and consisted out of denser particles, which were larger in size as compared with the P1 sample with a hexagonal shape. The particles sizes were not uniform and varied from 100 nm to 1 μm. The image of the highest magnification of sample P2 indicated towards small rods within the larger particles that were attached to the hexagonal particles, which offer more space and would be beneficial for gas sensing applications. The SEM images of sample P3 show an even more dense morphology due to the high thermal energy and particles were uniform in size and shape. The most of the CdO : ZnO particles of P4 were smaller in size and were found in bunches. This analysis clearly shows a formation of CdO : ZnO particles with hexagonal shape and the shape of the particles has deteriorating and formed bunches due to higher available thermal energy at the higher annealing temperatures. Umar *et al.*^21^ reported the sensing properties of the hexagonal nanocone structures, the advantage of grain boundaries or voids for the recombination and decrease charge transportation.

**Fig. 4:**
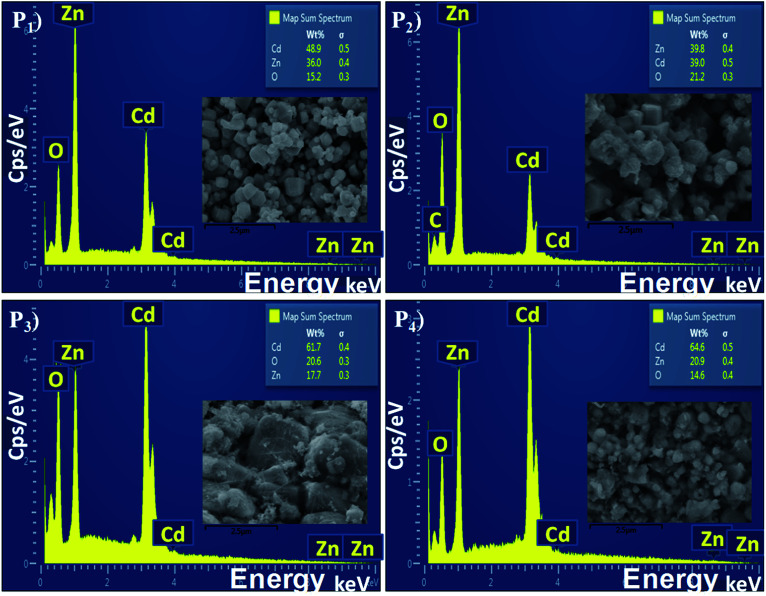
The energy dispersive X-ray spectra of the CdO : ZnO NPs annealed at 450 °C (P1), 500 °C (P2), 550 °C (P3) and 600 °C (P4), the SEM images are shown in the inset of every spectrum.

**Fig. 5:**
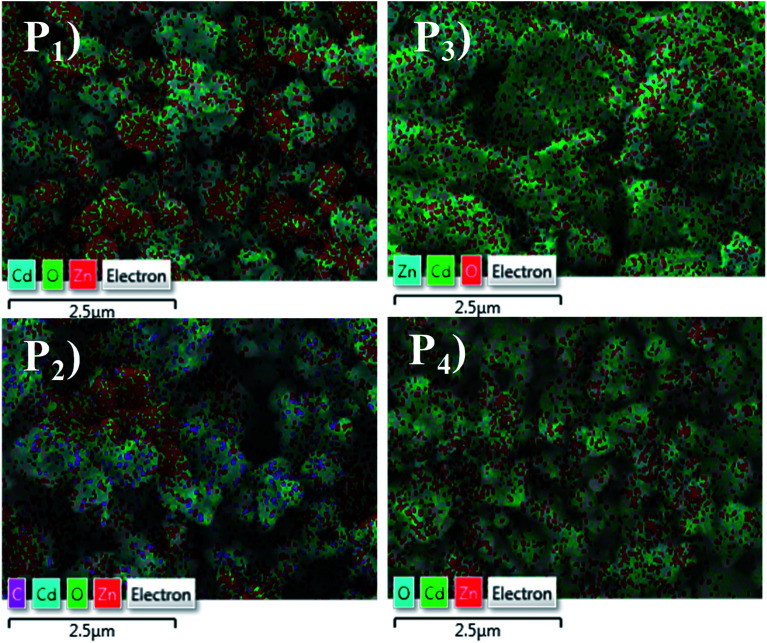
The energy dispersive X-ray mapping micrographs of CdO : ZnO NPs of the samples annealed at 450 °C (P1), 500 °C (P2), 550 °C (P3) and 600 °C (P4) with the different colour coding for the corresponding elements.

The elemental analysis of the CdO : ZnO particles were determined by EDX spectroscopy and are shown in Fig. 4 with the corresponding SEM images as insets. All the CdO : ZnO particles were of the same 3 : 1 ratio of cadmium and zinc, the wt% of the Cd and Zn was not constant in all the annealed samples (P1–P4). The density of Cd was found to be greater than Zn. The sample P2 shows a weak peak corresponding to carbon (C) energy which is due to the precursor acetates of Cd and Zn. For the detailed study of the elemental distributions the EDX mappings of Cd, Zn and oxygen (O) are shown in Fig. 5. The mapping images were taken by recording the energy *L*_α_ lines for Cd and Zn, while *K*_α_ was recorded for oxygen. These images are displaying the simultaneous distribution of Cd and Zn along with oxygen.

### 3.3 *I*–*V* characterization

**Fig. 6:**
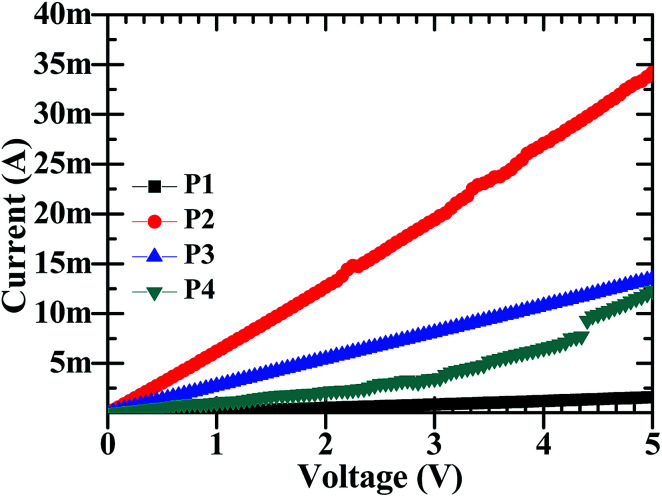
The *I*–*V* plots of the CdO : ZnO NCs (P1–P4) at room temperature (30 °C).

The electrochemical sensors work on the basic principle that a change in electrical resistance of the device with a changing physical condition *i.e.* Ohmic principle, occurs. It is therefore required to understand the DC electrical behaviour. The DC-electrical conductivity of CdO : ZnO NPs was investigated by *I*–*V* characteristics using a two probe method and is shown in Fig. 6, respectively. All the particles showed a linear variation in current with a change in the applied voltage in the range 0 to 5 V. The highest electrical conductivity was found for the NPs annealed at 500 °C, while the minimum was at 450 °C and the other two have intermediate conductivity. The electrical resistance of P1, P2, P3 and P4 NPs pellets were 4.55, 0.161, 0.366 and 0.926 kΩ, respectively at room temperature and exhibited good electrical conductivity. This shows that the electrical conductivity is a strong function of ATs, which is attributed to the structural properties. The electrical conductivity shows a linear relation with crystallite size and it's also influenced the defects in the crystal lattice.^7^

### 3.4 Oxygen sensing

**Fig. 7:**
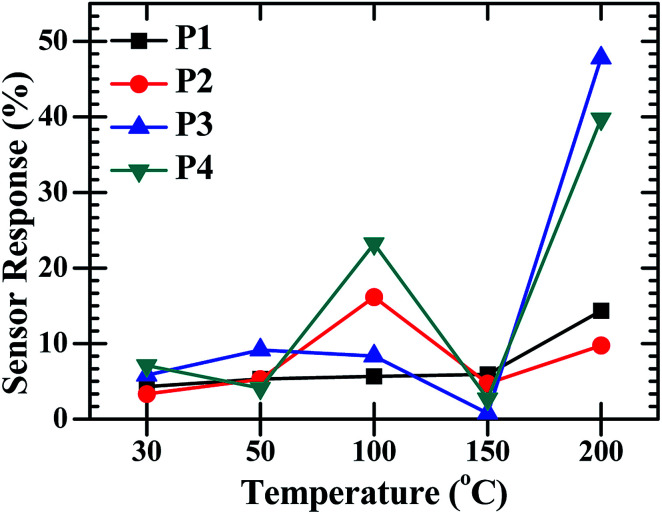
The O_2_ gas sensor response of the CdO : ZnO NCs annealed at different annealing temperatures (P1–P4) as a function of operating temperature with constant concentration of 450 sccm.

**Fig. 8:**
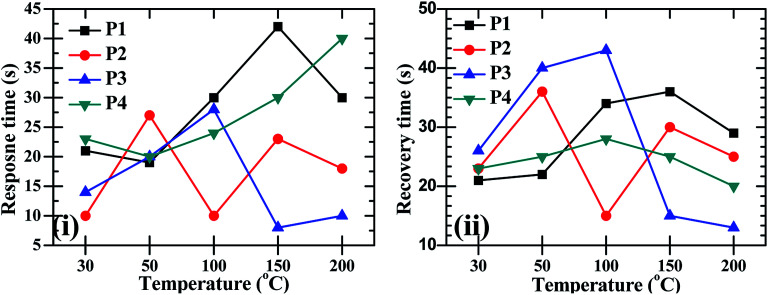
The O_2_ gas sensor response and recovery times of CdO : ZnO NCs annealed at different annealing temperatures (P1–P4) as a function of operating temperature.

**Fig. 9:**
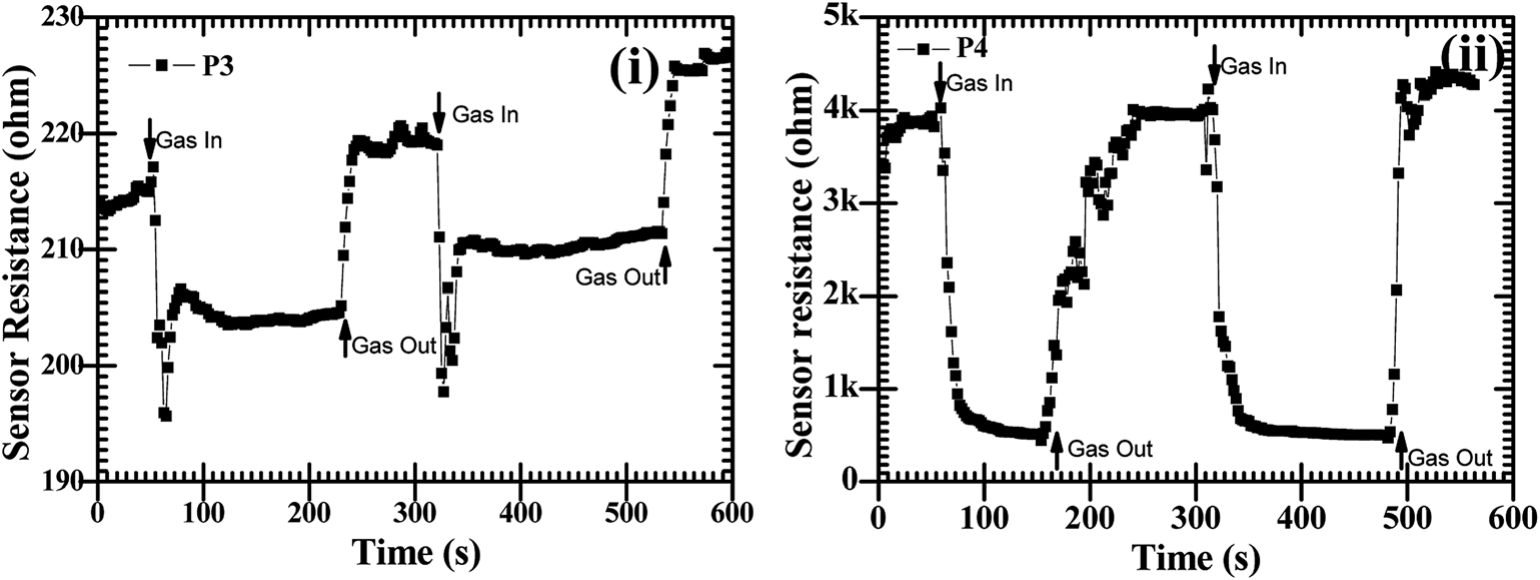
The O_2_ gas sensor response curves at operating temperature of 200 °C with a constant concentration of 450 sccm for CdO : ZnO NPs annealed at 550 °C, P3 (i) and 600 °C, P4 (ii).

After the confirmation of the formation of the CdO : ZnO hexagonal particles by the structural and elemental studies for the application of the hexagonal, the sensing experiments were performed for O_2_ gas in the temperature range 30–200 °C with a constant concentration of 450 sccm. The sensors showed a sensing response for only O_2_ gas as shown in Fig. 7. Semiconductor based electrochemical sensor responses are a strong function of operating temperature, so the CdO : ZnO sensor response varied with operating temperature and the highest response was found for the maximum operating temperature (200 °C). The sensor response was recorded at operating temperatures of 30, 50, 100, 150 and 200 °C. The maximum sensor response at operating temperature 30, 50, 100 and 150 °C was found to be 7% for P4, 9% for P3, 23% for P4, 5% for P1, respectively and the optimized results were obtained for sample P3 with a 47% sensor response and 39% for P4 at an operating temperature of 200 °C. Fig. 7 shows the low sensing response at the lower temperature range and the lowest response was found at 150 °C. The sensing response of a metal oxide based sensor strongly depends on operating temperature. Metal oxide sensing depends on the speed of the chemical reaction on the sensor surface, the speed of diffusion of the target gas molecules to that surface and the electron density of the sensor. These three are a function of temperature. At the lower operating temperature the speed of the chemical reaction is dominating and at the higher temperature the rate of diffusion takes part. The sensitivity is proposal to these two parameters and inversely proportional to the electron density. The lower sensing responses at 150 °C indicating the unbalance of the speed of reaction and the rate of diffusion of the gas molecules. The CdO : ZnO particles annealed at 550 °C and 600 °C showed good sensing response as compared to the other two samples, which were annealed at 450 °C and 500 °C, the possible reason is discussed in the next section. These sensing responses were rapid sensor responses with 10to 43 s response times and similarly recovery times of 13 s to 45 s. The variations of response and recovery times are shown in Fig. 8(i and ii). The response and recovery times were higher for the higher sensor response. This should be due to the sluggish surface reactions consisting of the adsorption and dissociation of O_2_ molecules.^22^ The sensing response curves of sample P3 and P4 are shown in Fig. 9, which show the variation in the electrical conductivity of the gas sensor in the presence of O_2_. These curves show a good stability of the sensor with a fast sensor response.

### 3.5 Sensing mechanism

The change in conductivity may be due to the chemical reaction between the interface of the sample surface and the adsorption of the oxygen ions (O_2_^−^, O^−^ or O^2−^ depending on the operating temperature).^19^ After accepting this well knows mechanism of gas sensing, the sensor response is discussed on the study of the present work. As we know, the sensitivity and fast response/recovery time depend on the surface morphology of the sample. The adsorption and dissociation of the gas molecules occur at the surface of the nanostructures and the high surface-to-volume ratio leads to active reaction sites. However, the tuneable pore size allows the gas molecules to easily penetrate and adsorb on the surface of the sensing materials, leading to a fast response and recovery as well as a high gas sensor response.^23^ The change in electrical resistance of the sensing material results due to the process of trapping or releasing back electrons.^24^ Target gas molecules expose to the sensor, charges transfer during the interaction between the chemisorbed oxygen vacancies and the tested gas molecules then change the surface resistance. When the surface depletion region is exposed to the target gas the gas reacts with O^−^ ions to release trapped electrons back into the conduction band of the semiconductor, resulting in a significant decrease in the resistance. The sensing response was found higher for sample P3 and P4 for the higher operating temperatures. From the analysis of the XRD, SEM, EDX, and *I*–*V* characteristics, the following conclusions can be formulated for the sensing response: (i) sample P3 and sample P4 were annealed at higher temperatures of 550 °C and 600 °C. These samples were found highly oriented cubic (111) and wurtzite (002) with small grains and the oxygen vacancies increased with annealing temperature. The exponential relation between vacancy formation and temperature is best describe by the Arrhenius equation. The well-known equation indicating the Arrhenius relation of vacancy formation as function of temperature is given by:*C*_v_ = exp(–*G*_F_/*kT*)where *C*_v_ is the concentration of vacancies, *G*_F_ the Gibbs energy, *k* Boltzman constant and *T* the absolute temperature. The XRD peaks positions were very nearly matched with the standard JCPDS for the P3 and P4 samples. (ii) The surface morphological images disclosed the shape of the CdO : ZnO particles. The P3 particles were separated to each other having a hexagonal shape, which offer more sites for the adsorption/desorption reaction, while for the P4 sample the particles were not in a regular size and amalgamate with small particles. The particle size of the P3 and P4 samples were found to be less as compared to the other two. (iii) The EDX spectra showed the higher cadmium percentage supports good sensitivity. The elemental mapping images of the CdO : ZnO particles keep up sensor response, the dots of Cd, Zn and O are speared in the whole images uniformly. (iv) Finally, the *I*–*V* characteristics showed an ohmic nature of the CdO : ZnO hexagonal particles with a good electrical conductivity, and results also revealed that the sensing properties should be independent to the variation of the electrical conductivity of the different samples. In view of real applications, a highly reliable gas sensor with little or no dependence on humidity or other parameters is extremely important for high-performance gas sensors. So, a highly conductive sensor is found more useful due to the little variation in conductivity as an effect of environmental parameters, which is negligible as compared to the variation in resistance due to target gas. The electron density of the sensor influenced the sensing response and is inversely proportional to the electron density. It must be pointed out that in CdO/ZnO heterostructures that the intrinsic Fermi level of the CdO is higher than that of ZnO.^25^ The potential energy of the electrons present in the CdO is there for higher than that in ZnO. Electrons can therefore be emitted from the CdO to the ZnO and as a result, an electron depletion region and an electron accumulation region are formed in the CdO and ZnO, respectively with the consequence of band banding that occurs in the process. This may be due to the discontinuity of the Fermi level along with the conduction and valence band, diffusion of carriers, lattice mismatch, and localized point defects. The so formed electron depletion region and electron accumulation region gives rise to an interface capacitance and the formation of a built in potential. Moreover the carrier concentration in the CdO is higher than that of ZnO, which leads to a significant diffusion of charge carriers in the conduction band of ZnO near the interface. Both the effects will contribute toward the formation of a conducting channel at the interface in the ZnO side, which may lead to big conductivity changes. Here all the samples were of the same volume ratio 3 : 1 (CdO : ZnO) so the influence on electron density only depend on the annealing temperature not the volume ratio. The electron density exponentially increased with operating temperature.

## 4. Conclusion

In summary, the sol–gel method was found a suitable chemical method to synthesis CdO : ZnO hexagonal particles consisting out of nanocrystallites. For gas sensing application, the CdO : ZnO hexagonal was suitable for the O_2_ gas sensing. The highly cubic-wurtzite composite polycrystalline lattice with the smaller grains have a good sensing response. The higher annealing temperature of 550 °C and 600 °C formed good small hexagonal particles as were seen in the SEM images. The sensing response against O_2_ gas with 450 sccm concentration was 47% and 39% at an operating temperature of 200 °C for the CdO : ZnO hexagonal particle, which were annealed at 550 °C and 600 °C, respectively. The sensor showed a rapid response and recovery speed with a good stability in the presence of the O_2_ gas.

## Conflicts of interest

There are no conflicts to declare.

## Supplementary Material
